# An open-label feasibility study of nintedanib combined with docetaxel in Japanese patients with locally advanced or metastatic lung adenocarcinoma after failure of first-line chemotherapy

**DOI:** 10.1007/s00280-018-3649-x

**Published:** 2018-08-03

**Authors:** Noboru Yamamoto, Hirotsugu Kenmotsu, Koichi Goto, Koji Takeda, Terufumi Kato, Masayuki Takeda, Hidehito Horinouchi, Isao Saito, Akiko Sarashina, Tetsuya Tanaka, Nassim Morsli, Kazuhiko Nakagawa

**Affiliations:** 1 Department of Thoracic Oncology, National Cancer Center Hospital, Tokyo, Japan; 2 Division of Thoracic Oncology, Shizuoka Cancer Center, Shizuoka, Japan; 3 Department of Thoracic Oncology, National Cancer Center Hospital East, Kashiwa, Japan; 4 Department of Medical Oncology, Osaka City General Hospital, Osaka, Japan; 5 Department of Respiratory Medicine, Kanagawa Cardiovascular and Respiratory Center, Yokohama, Japan; 6 Department of Medical Oncology, Kindai University Faculty of Medicine, Osaka, Japan; 7 Nippon Boehringer Ingelheim, Tokyo, Japan; 8 Nippon Boehringer Ingelheim, Kobe, Hyogo Japan; 9 EPS Corporation, Tokyo, Japan; 10 AstraZeneca Pharmaceuticals LP, Cambridge, UK

**Keywords:** Adenocarcinoma, Docetaxel, Japanese, Nintedanib, Non-small cell lung cancer

## Abstract

**Purpose:**

This open-label feasibility study assessed the tolerability of nintedanib 200 mg in combination with docetaxel 75 mg/m^2^ as a starting dose in Japanese patients with a body surface area (BSA) < 1.5 m^2^ and locally advanced or metastatic lung adenocarcinoma.

**Methods:**

Eligible patients received docetaxel 75 mg/m^2^ every 21 days and nintedanib administered at 200 mg twice daily (bid), starting on day 2 of each cycle. Treatment was continued until disease progression or undue toxicity. The primary endpoint was the number of patients experiencing dose-limiting toxicities (DLTs) in cycle 1 (days 1–21).

**Results:**

Of 10 treated patients, 2 patients (20%) experienced DLTs during cycle 1. These DLTs were grade 3 liver enzyme elevations [alanine aminotransferase (2 patients) and aspartate aminotransferase (2 patients)], and grade 2 hyperbilirubinemia (1 patient). Nine patients met the predefined criteria for nintedanib 200 mg bid plus docetaxel 75 mg/m^2^ to be considered a tolerable starting dose. All patients experienced ≥ 1 adverse event (AE) during the treatment period (all drug-related), but no patients experienced AEs that led to discontinuation of nintedanib. Of the five serious AEs reported during treatment, none were drug-related. There was no apparent effect of nintedanib on the pharmacokinetics of docetaxel. The objective response and disease control rates were 40 and 70%, respectively.

**Conclusion:**

Nintedanib 200 mg bid plus docetaxel 75 mg/m^2^ is a tolerable starting dose in Japanese patients with a BSA < 1.5 m^2^ with locally advanced or metastatic lung adenocarcinoma.

**ClinicalTrials.gov number:**

NCT02300298.

## Introduction

Prior to 2016, effective second-line treatment options in non-small cell lung cancer (NSCLC), particularly in patients with adenocarcinoma who are not eligible for epidermal growth factor receptor (EGFR)- or anaplastic lymphoma kinase (ALK)-targeted therapy, were limited [1, 2]. Despite recent Food and Drug Administration approval of the immunotherapies nivolumab, pembrolizumab, and atezolizumab in this setting, there remains a clinical need for treatment options with better efficacy than docetaxel in patients with aggressive disease whose tumors have low or absent programmed death-ligand 1 expression [3–6].

Nintedanib is an oral, triple angiokinase inhibitor that targets vascular endothelial growth factor (VEGF) receptors 1–3, fibroblast growth factor receptors 1–3, and platelet-derived growth factor receptors α/β. In addition, nintedanib inhibits receptor kinases of RET, Flt3, and the Src family [7].

Nintedanib, in combination with docetaxel, is approved in the European Union and other countries for the treatment of patients with locally advanced, metastatic, or locally recurrent NSCLC of adenocarcinoma histology after first-line chemotherapy [8, 9]. The efficacy and safety of nintedanib combined with docetaxel was confirmed in the phase III LUME-Lung 1 trial, in which the median overall survival of patients with adenocarcinoma who received docetaxel 75 mg/m^2^ plus nintedanib 200 mg twice daily (bid) was significantly longer than that in patients who received docetaxel plus placebo [10]. This treatment is, therefore, considered to be an effective second-line option for patients with advanced lung adenocarcinoma after failure of platinum-based therapy [10].

Phase I trials of nintedanib monotherapy have reported a difference between Japanese and European patients in the maximum tolerated dose (MTD) of nintedanib [11, 12]. Although liver enzyme elevations were among the dose-limiting toxicities (DLTs) of nintedanib in both Japanese and European patients, they occurred at a greater frequency in Japanese patients who were treated with the same doses, a finding that led to a difference in the MTD of nintedanib between European (250 mg bid) and Japanese (200 mg bid) patients [11, 12]. In a previous pharmacokinetic (PK) study, the authors noted that there was no difference in the reported PK profile of nintedanib between Japanese and European patients [11, 12], but that patient body size may affect the frequency of adverse events (AEs) [11, 12].

A phase I, open-label trial in Japanese patients with advanced NSCLC who received docetaxel 60 or 75 mg/m^2^ (on day 1) plus nintedanib 100, 150, or 200 mg bid (on days 2–21) reported that the MTD of nintedanib in combination with docetaxel 75 mg/m^2^ was 200 mg bid in patients with a body surface area (BSA) ≥ 1.5 m^2^, but 150 mg bid in patients with a BSA < 1.5 m^2^ [13]. This was because all patients with a BSA < 1.5 m^2^ who were treated with nintedanib 200 mg bid plus docetaxel 60 mg/m^2^ experienced DLTs during cycle 1, whereas only 1 of 6 patients with a BSA < 1.5 m^2^ who were treated with nintedanib 150 mg bid plus docetaxel 75 mg/m^2^ experienced DLTs during cycle 1. Given this, the lower nintedanib dose level (150 mg bid) was identified as the MTD in these patients. However, the majority of DLTs were dose-dependent liver enzyme elevations [alanine aminotransferase (ALT), gamma-glutamyltransferase (GGT), and aspartate aminotransferase (AST)] that were reversible with dose reduction and discontinuation [13]. Therefore, nintedanib 200 mg bid plus docetaxel 75 mg/m^2^, which had not been evaluated previously, was considered to be a potential starting dose in patients with a BSA < 1.5 m^2^ that warranted further investigation.

Consequently, we carried out an open-label, feasibility study to assess the tolerability of nintedanib 200 mg bid in combination with docetaxel 75 mg/m^2^ as a starting dose in Japanese patients with a BSA < 1.5 m^2^. Patients had locally advanced or metastatic lung adenocarcinoma that had progressed after first-line, platinum-based chemotherapy. The primary endpoint of the study was the number of patients experiencing a DLT during cycle 1.

## Materials and methods

### Study design and patients

This study was an open-label feasibility trial (phase Ib) in 10 Japanese patients. The study was conducted at 6 centers in Japan.

Eligible patients were ≥ 20 years old, with a BSA < 1.5 m^2^ at screening and histologically/cytologically confirmed locally advanced or metastatic lung adenocarcinoma after the failure of first-line platinum-based chemotherapy. Patients had a life expectancy of ≥ 3 months and an Eastern Cooperative Oncology Group performance status of 0 or 1 at screening. Patients who had received more than 1 prior line of chemotherapy for advanced or metastatic NSCLC were excluded from the study, as were patients who had received previous therapy with other VEGF or VEGF receptor inhibitors (other than bevacizumab) for the treatment of NSCLC at any time. Patients who had received other investigational drugs, chemotherapy, hormone therapy, immunotherapy, or monoclonal antibodies within 4 weeks of the start of the study, and those who had received molecular targeted therapy (including EGFR tyrosine kinase inhibitors and ALK inhibitors) within 2 weeks of the start of the study were also excluded.

All patients provided written informed consent. The study complied with the Declaration of Helsinki and was carried out in accordance with good clinical practice or regulatory guidelines and relevant local legislation. The protocol was approved by the independent ethics committee or institutional review boards at each center. An independent safety review committee continually monitored the safety of all patients taking part in the study.

### Procedures

All patients received docetaxel 75 mg/m^2^ by intravenous infusion over 1 h every 21 days with appropriate previous and concomitant medication, and nintedanib capsules were administered as an oral daily dose of 400 mg (200 mg in the morning and 200 mg in the evening after meals) starting on day 2 of each cycle.

There was no prespecified maximum number of treatment cycles; treatment was continued as long as patients did not meet any of the treatment discontinuation criteria, such as disease progression or undue toxicity. In the case of related AEs, 2 dose reduction steps were permitted for both docetaxel and nintedanib. Docetaxel could be reduced from 75 to 60 mg/m^2^ for the first dose reduction and from 60 to 50 mg/m^2^ for the second dose reduction. Nintedanib could be reduced from 200 to 150 mg bid for the first dose reduction and from 150 to 100 mg bid for the second dose reduction. No dose increases were allowed after a dose reduction for either nintedanib or docetaxel.

Patients who had to discontinue docetaxel for reasons other than disease progression were allowed to continue treatment with nintedanib monotherapy. Patients were monitored at regular intervals for evaluation of safety, assessment of compliance, recording of AEs, and additional examinations. Tumor response was assessed using Response Evaluation Criteria in Solid Tumors version 1.1, at baseline and on day 1 of every odd cycle after cycle 3 until disease progression. AEs, classified according to the Common Terminology Criteria for Adverse Events (CTCAE) version 3.0, were recorded during the study period. A serious AE (SAE) was defined as any AE that resulted in death, was immediately life-threatening, resulted in persistent or significant disability/incapacity, needed admission to hospital or prolonged admission to hospital, or was a congenital anomaly or birth defect. Other events were deemed serious if, on the basis of appropriate medical judgment, the event might jeopardize the patient and need medical or surgical intervention to prevent one of the other outcomes listed in the above definition. Patients were monitored for AEs throughout the study according to the visit schedule defined in the protocol.

### Evaluation of appropriate starting dose

The appropriateness of nintedanib 200 mg bid plus docetaxel 75 mg/m^2^ as a starting dose was evaluated by the safety review committee comprising 3 physicians with considerable experience in the management of NSCLC in the clinical trial setting. This committee reviewed data from study participants according to the following predefined criteria: (1) a patient who completed cycle 1 without any DLTs; (2) a patient who experienced a DLT of CTCAE grade ≥ 3 AST and/or ALT increase, or CTCAE grade 2 AST and/or ALT increase in conjunction with CTCAE grade ≥ 1 total bilirubin in cycle 1 but could be retreated with the combination of nintedanib 150 mg plus docetaxel 75 mg/m^2^ and completed cycle 2 without any further DLT.

If a patient experienced DLTs other than CTCAE grade ≥ 3 AST and/or ALT increase, the starting dose was not considered to be appropriate, even if nintedanib could be administered at a reduced dose. If ≥ 4 patients experienced DLTs other than CTCAE grade ≥ 3 AST and/or ALT increase in cycle 1 during the enrollment period, the investigators were to halt patient recruitment and discuss next steps with the safety review committee.

### Outcomes

The primary endpoint was the number of patients experiencing DLTs in cycle 1 (days 1–21), according to the CTCAE version 3.0. DLTs are defined in Table 1.

**Table 1:** Definitions of dose-limiting toxicities

Criteria
CTCAE grade ≥ 3 non-hematologic toxicity (except transient electrolyte abnormality and isolated increase of GGT) Gastrointestinal toxicity (e.g., nausea, vomiting, diarrhea, abdominal pain) despite adequate supportive care
CTCAE grade 4 hematological toxicity Decreased neutrophil count (not associated with fever ≥ 38.5 °C) for > 7 days, despite adequate supportive treatment (e.g., G-CSF) Decreased WBC count for > 7 days, despite adequate supportive treatment (e.g., G-CSF)
CTCAE grade 4 febrile neutropenia associated with fever ≥ 38.5 °C
CTCAE grade ≥ 2 ALT and/or increased AST in conjunction with CTCAE grade ≥ 2 increase of total bilirubin
Inability to resume nintedanib dosing within 14 days after stopping because of toxicity

*ALT* alanine aminotransferase, *AST* aspartate aminotransferase, *CTCAE* Common Terminology Criteria for Adverse Events, *GGT* gamma-glutamyltransferase, *G-CSF* granulocyte-colony stimulating factor, *WBC* white blood cell

Secondary endpoints included PK parameters for nintedanib and docetaxel: maximum measured concentration of the analyte in plasma (*C*_max_), area under the concentration–time curve of the analyte in plasma over the time interval from 0 to time of the last quantifiable plasma concentration (AUC_0−tz_), and area under the concentration–time curve of the analyte in plasma over the time interval from 0 extrapolated to infinity (AUC_0−∞_).

Safety endpoints included the number of patients with AEs according to CTCAE (version 3.0) for overall AEs, SAEs, drug-related AEs, AEs leading to dose reduction or treatment discontinuation, AEs of special interest, and changes in laboratory parameters. The objective response rate (percentage of patients whose best overall response was complete response or partial response) and disease control rate (percentage of patients whose best overall response was complete response, partial response or stable disease) were evaluated as further endpoints.

### Statistical analysis

Descriptive statistics only were used in this study; no statistical model was adopted. The analysis sets were defined for the analysis of safety, efficacy, and PK. The treated set included all patients who received study medication and were documented to have taken ≥ 1 dose of study treatment (docetaxel or nintedanib); this patient set was used for the analysis of safety and efficacy except for the primary endpoint. The feasibility set included all patients who were included in the treated set, except any replaced patients; this patient set was used for the analysis of the primary endpoint. The PK set included all patients in the treated set who had ≥ 1 available valid drug plasma concentration; this patient set was used for the analysis of PK.

The incidence of DLTs graded according to CTCAE version 3.0 was evaluated; the frequency, severity, and causal relationship of AEs were tabulated by system organ class and preferred term after coding according to version 18.1 of the Medical Dictionary for Regulatory Activities. AEs and laboratory parameters were graded according to the CTCAE version 3.0. PK parameters were calculated by standard non-compartmental methods. Best overall response was tabulated in frequency (%).

## Results

### Patient demographics

**Fig. 1:**
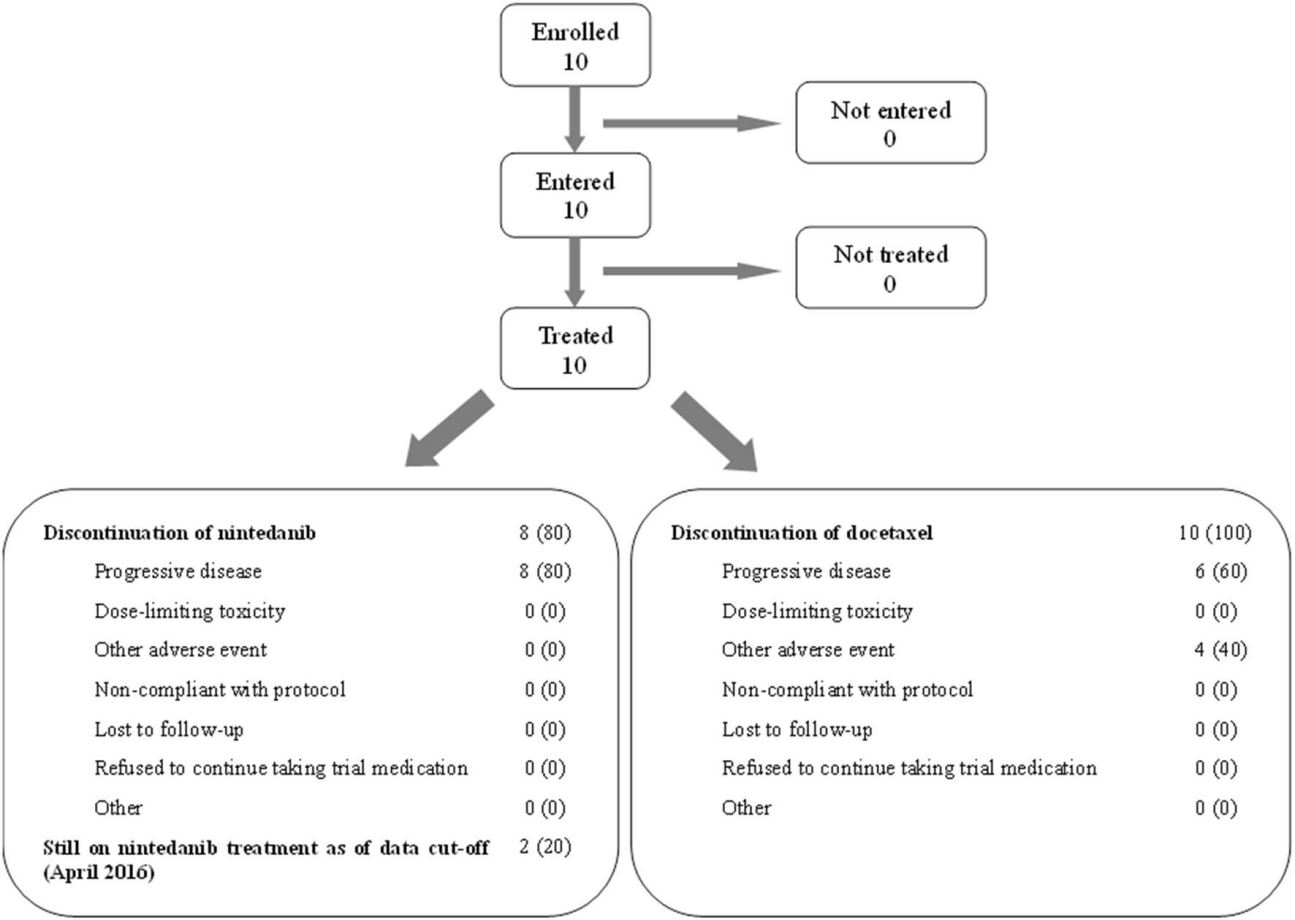
Patient disposition

A total of 10 patients (2 male and 8 female) with locally advanced or metastatic lung adenocarcinoma were enrolled between January 2015 and September 2015. As of the data cut-off (April 2016), 2 patients were still receiving treatment with nintedanib monotherapy (Fig. 1). The reason for discontinuation of nintedanib was progressive disease in all 8 patients. The most common reason for discontinuation of docetaxel was progressive disease [6 patients (60%)], followed by other AEs [4 patients (40%)].

The demographics and clinical characteristics of the patients are listed in Table 2. The median age of patients was 69 years and the median BSA was 1.45 m^2^. All 10 patients had previously been treated with platinum-based chemotherapy: 7 patients with cisplatin and 3 patients with carboplatin. The majority of patients were on doublet combination treatment that included pemetrexed (7 patients); other patients were on doublet combination treatment that included vinorelbine (2 patients), or quartet combination treatment, including paclitaxel, bevacizumab, and GDC0941 (1 patient).

**Table 2:** Patient demographics and baseline disease characteristics

	Nintedanib 200 mg with docetaxel 75 mg/m^2^
Number of patients, *N* (%)	10 (100.0)
Gender, *n* (%)	
Male	2 (20.0)
Female	8 (80.0)
Age (years)	
Median (min–max)	69 (55–75)
BSA (m^2^)	
Median (min–max)	1.45 (1.41–1.48)
Body weight (kg)	
Median (min–max)	48.40 (40.8–51.9)
Height (cm)	
Median (min–max)	155.5 (151–165)
Smoking status, *n* (%)	
Never smoked	8 (80.0)
Ex-smoker	2 (20.0)
Currently smokes	0 (0.0)
ECOG PS, *n* (%)	
0	4 (40.0)
1	6 (60.0)
Metastatic disease at screening, *n* (%)	
No	0 (0.0)
Yes	10 (100.0)
Number of metastatic sites	
Median (min–max)	1.00 (1.0–3.0)
Best response to first-line, platinum-based chemotherapy, *n* (%)	
CR/PR/SD	7 (70.0)
PD	1 (10.0)
Not applicable/unknown	2 (20.0)
EGFR mutation status, *n* (%)	
Negative	6 (60.0)
Positive	4 (40.0)
ALK mutation status, *n* (%)	
Positive	0 (0.0)
Negative	7 (70.0)
Unknown	3 (30.0)

*ALK* anaplastic lymphoma kinase, *BSA* body surface area, *CR* complete response, *ECOG PS* Eastern Cooperative Oncology Group performance status, *EGFR* epidermal growth factor receptor, *PD* progressive disease, *PR* partial response, *SD* stable disease

### Exposure to study drug

Overall, the median duration of exposure to trial medication (nintedanib or docetaxel) was 130.0 days (range 57–428 days). All patients (100%) received ≥ 1 dose of nintedanib. The median duration of exposure to nintedanib was 129 days (range 56–427 days) and the median dose intensity was 92.8% (range 49–100%). Of the 10 patients treated, 5 had a dose reduction of nintedanib from 200 to 150 mg bid, and 1 patient had a second dose reduction to 100 mg bid. Time to the first dose reduction ranged from 15 to 128 days (median 46 days). All patients (100%) received ≥ 1 dose of docetaxel. The median number of docetaxel cycles was 5.5 (range 3–16) and the median dose intensity was 87.3% (range 78–100%). Of the 10 treated patients, 8 patients had a dose reduction of docetaxel from 75 to 60 mg/m^2^ and 4 patients had a second dose reduction from 60 to 50 mg/m^2^. Time to the first dose reduction ranged from 28 to 204 days (median 47.5 days).

### DLTs

In cycle 1, DLTs were reported in 2 patients (20%) (Table 3). These DLTs were CTCAE grade 3 liver enzyme [ALT (2 patients) and AST (2 patients)] elevations, and CTCAE grade 2 hyperbilirubinemia (1 patient). Of the 2 patients with DLTs in cycle 1, patient 1 (who had CTCAE grade 3 increased ALT and CTCAE grade 3 increased AST in conjunction with CTCAE grade 2 hyperbilirubinemia in cycle 1) reported additional DLTs by the end of cycle 2 (CTCAE grade 3 increased ALT, CTCAE grade 3 increased AST, and CTCAE grade 2 hyperbilirubinemia); this patient required a second dose reduction of nintedanib (100 mg bid), as well as a dose reduction of docetaxel (60 mg/m^2^). Patient 1 was continuing nintedanib treatment at 100 mg bid at the data cut-off (April 2016). Patient 2 (who had experienced CTCAE grade 3 increased ALT and CTCAE grade 3 increased AST) was able to be retreated with a reduced nintedanib dose of 150 mg bid in combination with docetaxel 75 mg/m^2^, and completed cycle 2 without further DLTs; they then went on to receive 6 cycles of combination therapy. Therefore, 9 of 10 patients included in this study met the predefined criteria for the dose of nintedanib 200 mg bid in combination with docetaxel 75 mg/m^2^ to be considered a tolerable starting dose. Eight patients completed cycle 1 without any DLTs, and 1 experienced DLTs but could be retreated with the combination of nintedanib 150 mg plus docetaxel 75 mg/m^2^ and completed cycle 2 without any further DLTs.

**Table 3:** Observed DLTs

Patient	Nintedanib dose at onset of AE (mg)	Grade and preferred term	Cycle
Patients who experienced DLTs in cycle 1 (with or without DLTs after cycle 1)			
1	200 bid	Grade 3 increase in ALT, grade 3 increase in AST and grade 2 hyperbilirubinemia	Cycle 1
	150 bid	Grade 3 increase in ALT, grade 3 increase in AST and grade 2 hyperbilirubinemia	After cycle 1
2	200 bid	Grade 3 increase in ALT and AST	Cycle 1
Patients who experienced DLTs only after cycle 1			
3	200 bid	Grade 3 increase in AST	After cycle 1
4	200 bid	Grade 3 increase in ALT	After cycle 1

*AE* adverse event, *ALT* alanine aminotransferase, *AST* aspartate aminotransferase, *bid* twice daily, *DLT* dose-limiting toxicity

An additional 2 patients reported DLTs occurring after cycle 1 (patients 3 and 4). Patient 3 reported CTCAE grade 3 increased AST and patient 4 reported CTCAE grade 3 increased ALT. In both patients, nintedanib was successfully re-introduced at 150 mg bid.

### Safety

All patients (10 patients, 100%) experienced ≥ 1 AE during the on-treatment period, all of which were considered to be drug related by the investigator (Table 4). The most common AEs were decreased neutrophil count and decreased white blood cell (WBC) count (10 patients each, 100%), alopecia (9 patients, 90%), increased ALT (8 patients, 80%), increased AST (7 patients, 70%), constipation, diarrhea, and nausea (5 patients each, 50%) (Table 4). The most common grade ≥ 3 AEs were decreased neutrophil count, decreased WBC count (10 patients each, 100%), increased ALT, increased AST (3 patients each, 30%), increased GGT, decreased lymphocyte count, and febrile neutropenia (2 patients each, 20%) (Table 4). Both cases of febrile neutropenia were grade 3.

**Table 4:** AEs that were reported for more than 1 patient

System organ class/preferred term	Nintedanib 200 mg with docetaxel 75 mg/m^2^	
	All grades *N* (%)	Grade 3/4 *N* (%)
Number of patients	10 (100.0)	10 (100.0)
Total with any AEs	10 (100.0)	10 (100.0)
Blood and lymphatic system disorders	2 (20.0)	2 (20.0)
Febrile neutropenia	2 (20.0)	2 (20.0)
Eye disorders	5 (50.0)	0 (0.0)
Lacrimation increased	2 (20.0)	0 (0.0)
Gastrointestinal disorders	9 (90.0)	0 (0.0)
Constipation	5 (50.0)	0 (0.0)
Diarrhea	5 (50.0)	0 (0.0)
Nausea	5 (50.0)	0 (0.0)
Vomiting	2 (20.0)	0 (0.0)
General disorders and administration-site conditions	8 (80.0)	0 (0.0)
Fatigue	4 (40.0)	0 (0.0)
Edema peripheral	3 (30.0)	0 (0.0)
Pyrexia	3 (30.0)	0 (0.0)
Malaise	2 (20.0)	0 (0.0)
Infections and infestations	6 (60.0)	0 (0.0)
Cystitis	2 (20.0)	0 (0.0)
Upper respiratory tract infection	2 (20.0)	0 (0.0)
Investigations	10 (100.0)	10 (100.0)
Neutrophil count decreased	10 (100.0)	10 (100.0)
WBC count decreased	10 (100.0)	10 (100.0)
ALT increased	8 (80.0)	3 (30.0)
AST increased	7 (70.0)	3 (30.0)
GGT increased	4 (40.0)	2 (20.0)
Blood alkaline phosphatase increased	2 (20.0)	0 (0.0)
Hemoglobin decreased	2 (20.0)	0 (0.0)
Lymphocyte count decreased	2 (20.0)	2 (20.0)
Metabolism and nutrition disorders	4 (40.0)	1 (10.0)
Decreased appetite	4 (40.0)	0 (0.0)
Musculoskeletal and connective tissue disorders	2 (20.0)	0 (0.0)
Myalgia	2 (20.0)	0 (0.0)
Nervous system disorders	5 (50.0)	0 (0.0)
Peripheral sensory neuropathy	4 (40.0)	0 (0.0)
Dysgeusia	3 (30.0)	0 (0.0)
Headache	2 (20.0)	0 (0.0)
Psychiatric disorders	3 (30.0)	0 (0.0)
Insomnia	3 (30.0)	0 (0.0)
Skin and subcutaneous tissue disorders	9 (90.0)	0 (0.0)
Alopecia	9 (90.0)	0 (0.0)
Palmar–Plantar erythrodysesthesia syndrome	2 (20.0)	0 (0.0)
Pruritus	2 (20.0)	0 (0.0)
Rash	2 (20.0)	0 (0.0)
Vascular disorders	4 (40.0)	1 (10.0)
Flushing	2 (20.0)	0 (0.0)

*AE* adverse event, *ALT* alanine aminotransferase, *AST* aspartate aminotransferase, *GGT* gamma-glutamyltransferase, *WBC* white blood cell

The highest CTCAE grades reported were grade 4 for 9 patients (90%) and grade 3 for 1 patient (10%). No fatal (grade 5) AEs were reported. No patients experienced AEs that led to discontinuation of nintedanib, whereas 4 patients experienced AEs leading to discontinuation of docetaxel.

AEs leading to dose reduction of nintedanib and docetaxel were reported for 5 (50%) patients and 8 (80%) patients, respectively. The most common AEs leading to dose reduction of nintedanib were increased ALT and increased AST (30% each); the most common AE leading to dose reduction of docetaxel was decreased neutrophil count (40%), followed by decreased WBC count and febrile neutropenia (20% each). A total of 5 SAEs were reported in 5 patients during treatment. None of the SAEs were considered to be drug related by the investigator and none led to dose reduction or discontinuation of study medication.

Overall, liver enzyme elevations of > 1 CTCAE grade from baseline were observed in 5 (50%) patients for AST, 6 (60%) patients for ALT, 7 (70%) patients for GGT, 1 (10%) patient for alkaline phosphatase, and 1 (10%) patient for total bilirubin. Combined liver transaminase and bilirubin elevations were reported for 1 (10%) of 10 treated patients; this patient (patient 1) was a potential Hy’s law case (alkaline phosphatase levels were consistently below 2 times the upper limit of normal and there was no alternative explanation for liver enzyme elevation). CTCAE grade 3 or 4 decreases in WBC count and neutrophil count were observed in all patients. Clinically significant values of laboratory tests were mainly attributed to hematologic and liver parameter-related side effects of the therapy; other significant values included low albumin (30%), high glucose (20%), high calcium, low phosphate, and high lactate dehydrogenase (10% each). No clinically significant values were observed for coagulation parameters.

### PK

After single oral administration of 200 mg nintedanib, the plasma concentration of nintedanib and its metabolite, BIBF 1202 ZW, reached its maximum, with median time of *C*_max_ (*t*_max_) values of 2.00 and 3.98 h, and then declined with a geometric mean half-life of 6.97 and 5.88 h, respectively. After single oral administration of 200 mg nintedanib, the plasma concentration of BIBF 1202 glucuronide reached its maximum, with a median *t*_max_ value of 9.97 h, and then declined slightly and remained at almost the same level up to 24 h after nintedanib administration.

Geometric mean values of the ratio of exposures (AUC_0−12_ and *C*_max_) of BIBF 1202 ZW relative to exposures of nintedanib were 1.05 and 0.847, respectively. Geometric mean values of the ratio of exposures for the second metabolite BIBF 1202 glucuronide relative to the exposures of nintedanib were 1.25 and 0.725, respectively. The geometric mean values of plasma exposure, measured by AUC_0−tz_, in cycle 1 were higher in patients with DLTs (*n* = 2) versus those without (*n* = 7). This trend could be observed for the free-base form of nintedanib (614 vs. 289 ng × h/mL, respectively), BIBF 1202 ZW (696 vs. 301 ng × h/mL, respectively), and BIBF 1202 glucuronide (1960 vs. 865 ng × h/mL, respectively). However, owing to the low number of patients, no firm conclusions about this observation can be reached.

After intravenous administration, the plasma concentration of docetaxel reached maximum at around the end of infusion and then declined, with a half-life of 20.7 h. Geometric mean values of AUCs in cycle 2 were comparable to those in cycle 1.

### Tumor response

Seven patients were evaluable for tumor response in the treated set (*n* = 10). In this patient set, the best response was a partial response in 4 patients (40%) and stable disease in 3 patients (30%). The objective response rate was 40% and the disease control rate was 70%. All patients with target lesions at baseline (7 patients) achieved disease control.

## Discussion

This open-label feasibility trial was conducted to determine the tolerability of nintedanib 200 mg bid in combination with docetaxel 75 mg/m^2^ as a starting dose in Japanese patients with a BSA < 1.5 m^2^ and locally advanced or metastatic lung after the failure of platinum-based chemotherapy.

This investigation into an appropriate starting dose was initiated given the clinical benefits of nintedanib combination therapy previously observed in patients with lung adenocarcinoma in the global phase III LUME-Lung 1 trial [10].

Using a similar definition of DLTs as per a previous phase I, open-label trial [13], DLTs were reported for 2 (20%) of 10 patients in cycle 1. DLTs were CTCAE grade 3 liver enzyme elevations (ALT and AST), in conjunction with CTCAE grade 2 hyperbilirubinemia in 1 patient. One of the 2 patients was able to be retreated with a reduced nintedanib dose of 150 mg bid in combination with docetaxel 75 mg/m^2^ and completed cycle 2 without any further DLT. The second patient, who had CTCAE grade 2 hyperbilirubinemia, required a second dose reduction of nintedanib (100 mg bid). In patients who reported DLTs, elevated indices of exposure of the free-base form of nintedanib and its metabolites relative to patients without DLTs were observed; however, it should be noted that the patient numbers were low and this trend would be expected given that increased drug exposure is associated with an increased severity of AEs [14].

Differences were observed in the proportion of DLTs reported in cycle 1 of our study (40%) and a previous phase I, open-label trial (100%), which had comparable numbers of patients with BSA < 1.5 m^2^; these minor differences can be attributable to the small sample size of patients in both studies.

Overall, the safety profile of the combination therapy was consistent with previous clinical trials with nintedanib and the known safety profile of docetaxel, with the most common drug-related AEs being myelotoxicity (neutropenia and leukopenia), liver enzyme elevations (increased AST and ALT), gastrointestinal AEs (diarrhea and nausea), and alopecia. Neutropenia and leukopenia were CTCAE grade 3 or 4 in all patients; however, these events were manageable with adequate supportive treatment or dose reduction of docetaxel, and no events lasted over 7 days to qualify as a DLT. A total of 4 patients had liver enzyme elevations of CTCAE grade 3. These events were accompanied by a moderate decrease in parameters associated with liver function (pre-albumin, albumin, and cholinesterase) or abnormality in abdominal imaging; however, all tended to be resolved after treatment interruption and were manageable. Treatment with nintedanib was successfully re-introduced at lower doses in all patients. Clinical laboratory safety parameter findings in this trial were consistent with the pattern of the observed AEs and were mainly attributed to liver parameter elevations associated with nintedanib and hematological side effects of docetaxel.

Although the starting dose of nintedanib 200 mg bid in combination with docetaxel 75 mg/m^2^ was considered to be well-tolerated in Japanese patients with a BSA < 1.5 m^2^, it should be noted that the frequency of patients who required ≥ 1 dose reduction of trial medications was high in this trial (nintedanib 50%, docetaxel 80%) compared to the adenocarcinoma subpopulation in the phase III LUME-Lung 1 trial (nintedanib 21.9%, docetaxel 16.9%) [10]. In our trial, geometric mean values of AUC for docetaxel were comparable in cycles 1 and 2, suggesting that there is no effect of nintedanib on docetaxel exposure in the applied treatment schedule in patients with a BSA < 1.5 m^2^. This is in agreement with previous clinical data in non-Japanese and Japanese patients [13, 15].

Overall, 4 (40%) of 10 patients achieved a partial response and all patients with target lesions achieved disease control. Taking into consideration the small sample size, these findings indicate a clinical benefit of nintedanib plus docetaxel in Japanese patients with lung adenocarcinoma after platinum-based chemotherapy, and are consistent with results from the phase III LUME-Lung 1 trial [10].

## Conclusion

Results from this open-label feasibility study demonstrated that nintedanib 200 mg bid in combination with docetaxel 75 mg/m^2^ in a 21-day cycle is a tolerable starting dose in Japanese patients with a BSA < 1.5 m^2^ and locally advanced or metastatic lung adenocarcinoma. In addition, preliminary efficacy analyses indicate that this dose exhibited antitumor activity.
